# Comparative Gut Microbiota of 59 Neotropical Bird Species

**DOI:** 10.3389/fmicb.2015.01403

**Published:** 2015-12-21

**Authors:** Sarah M. Hird, César Sánchez, Bryan C. Carstens, Robb T. Brumfield

**Affiliations:** ^1^Museum of Natural Science, Louisiana State UniversityBaton Rouge, LA, USA; ^2^Department of Biological Sciences, Louisiana State UniversityBaton Rouge, LA, USA; ^3^Department of Evolution, Ecology and Organismal Biology, Ohio State UniversityColumbus, OH, USA

**Keywords:** Neotropical birds, gut microbiota, V6, host-associated microbiota, evolution

## Abstract

The gut microbiota of vertebrates are essential to host health. Most non-model vertebrates, however, lack even a basic description of natural gut microbiota biodiversity. Here, we sampled 116 intestines from 59 Neotropical bird species and used the V6 region of the 16S rRNA molecule as a microbial fingerprint (average coverage per bird ~80,000 reads). A core microbiota of Proteobacteria, Firmicutes, Bacteroidetes, and Actinobacteria was identified, as well as several gut-associated genera. We tested 18 categorical variables associated with each bird for significant correlation to the gut microbiota; host taxonomic categories were most frequently significant and explained the most variation. Ecological variables (e.g., diet, foraging stratum) were also frequently significant but explained less variation. Little evidence was found for a significant influence of geographic space. Finally, we suggest that microbial sampling during field collection of organisms would propel biological understanding of evolutionary history and ecological significance of host-associated microbiota.

## Introduction

Gut microbiota are an essential component of vertebrate health. Microbes provide many necessary functions for the host organism, including aiding digestion, vitamin synthesis, protection against pathogens, training the immune system and organ development (Qin et al., 2010; Diaz Heijtz et al., 2011; Al-Asmakh et al., 2014). The gut microbiota is one of the most densely populated natural environments known (Whitman et al., 1998), possibly composed of thousands of species (Xu and Gordon, 2003). Generally speaking, gut microbiota communities tend to be more similar between more similar hosts, although the specific members of the microbiota can vary significantly between hosts of the same species (Eckburg et al., 2005; Hird et al., 2014) and even between identical twins (Turnbaugh et al., 2010). The microbiota may have an influence above the level of the individual, as they can affect mate choice (Sharon et al., 2010) and cause hybrid inviability (Brucker and Bordenstein, 2013). Understanding the role of the microbiota in evolution is a major outstanding question and the subject of much ongoing research.

Birds are a globally distributed class of vertebrates. The bird lineage is thought to be approximately 150 million years old and has likely been in symbiotic relationships with microorganisms the entire time. Birds live on every continent and exhibit extreme morphological and ecological diversity, much of which is centered in the Neotropics (Jenkins et al., 2013). Pertinent to gut microbiota, bird diets range from robust and opportunistic to strictly carrion feeding (i.e., vultures) to nectar feeding (i.e., hummingbirds) to folivorous (i.e., hoatzin) with corresponding variation in their intestinal morphology. Most avian gut microbiota studies have looked at one or a few host species; from these studies we know that bird gut microbiota are influenced by host genetics and evolutionary history (Banks et al., 2009; Dewar et al., 2013) as well as by ecological factors, such as dietary specialization (Roggenbuck et al., 2014). The gut communities can also change as a result of seasonal dietary fluctuations (Wienemann et al., 2011).

Modern molecular techniques provide an insight into the biodiversity contained within the guts of birds. Firmicutes, Proteobacteria, and Actinobacteria tend to dominate avian gut samples (summarized in Waite and Taylor, 2014), although captivity status can have a significant effect on microbial composition (Xenoulis et al., 2010; Wienemann et al., 2011; Kohl, 2012). Most gut microbiota studies have not included physical space as a variable that may be contributing to microbial variation in host gut communities. Geographic distance is associated with gut microbiota similarity (Yatsunenko et al., 2012) and, generally speaking, bacterial communities that are geographically closer are more similar than communities more distant (Green and Bohannan, 2006). Avian gut microbiota have shown conflicting results regarding the importance of geographic distance; whereas some studies have found it to be associated with the gut microbiota (Hird et al., 2014), others have looked but found no such evidence (Banks et al., 2009).

Large comparative studies inform us about the relationship between higher taxonomic classes and broader ecological groups. In mammals, such studies have found that host taxonomy is strongly associated with gut microbiota communities (Ley et al., 2008a) as is dietary specialization (Ley et al., 2008b; Muegge et al., 2011). Diet seems to be the most important factor across insects (Anderson et al., 2012; Colman et al., 2012) and environmental variables have the most influence on gut microbiota in fishes (Sullam et al., 2012), although taxonomy may also have a role. A recent meta-analysis of all previously published bird gut microbiota studies found that taxonomy of the bird had a major influence on the composition of the gut microbes (Waite and Taylor, 2014). Understanding how various host and environmental factors interact to shape the gut microbiota is a major goal of modern microbial ecology and evolution.

A molecular survey of the gut microbiota is a basic biodiversity measure that is lacking for the vast majority of wild bird species. Our primary aim for the current study was to create a microbial catalog for the gut microbiota diversity found in 59 Neotropical bird species sampled in the wild. Second, we test for taxonomic and ecological associations between bird host and gut microbiota. Finally, we tested for a geographic signal in our dataset, which was gathered from 12 localities across Costa Rica and Peru.

## Materials and methods

### Sampling

**Figure 1:**
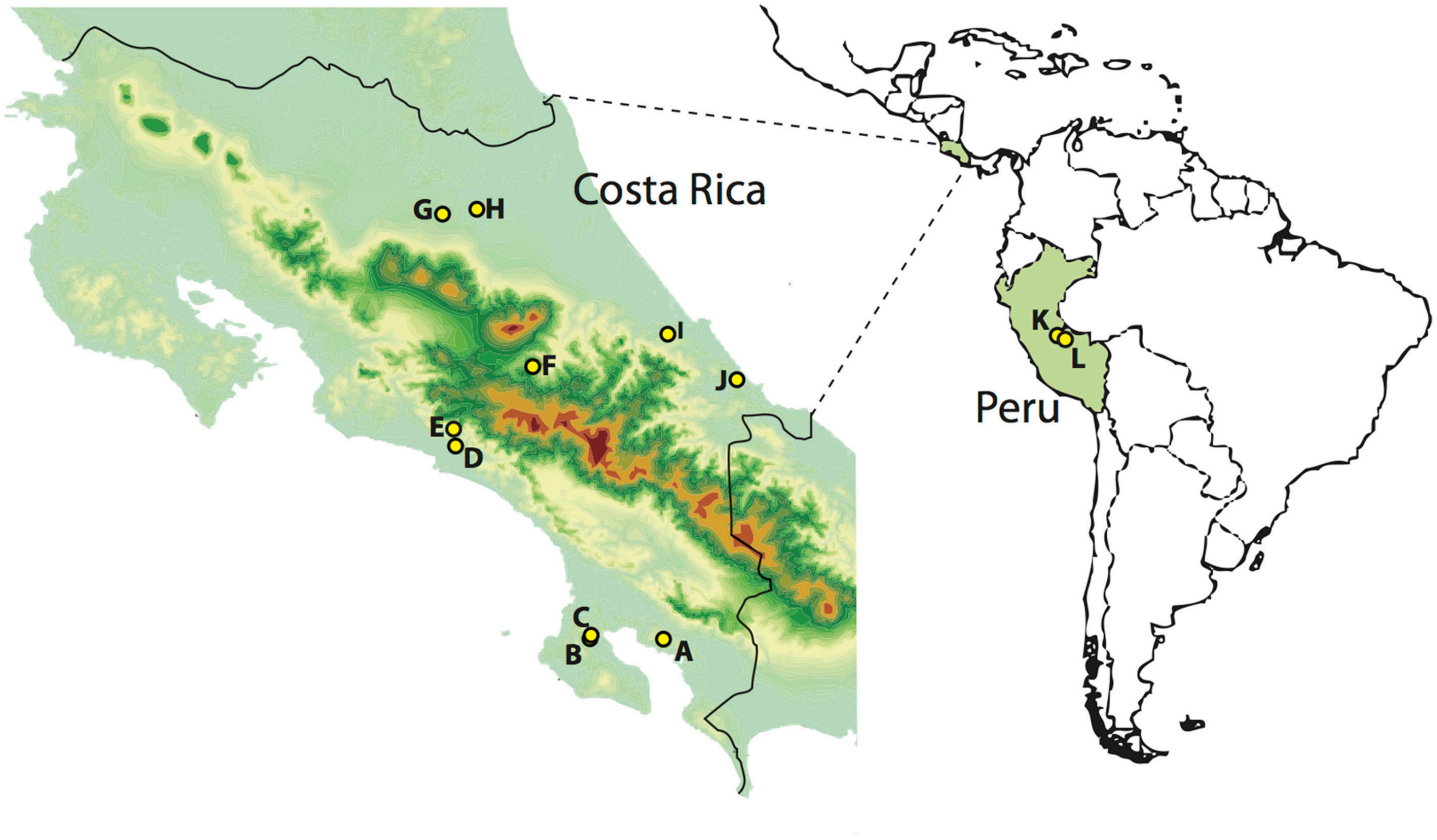
**Sampling localities in Costa Rica (left) and Peru (right)**. A, Piedras Blancas; B, Los Charcos; C, Golfo Dulce; D, Londres; E, Santa Juana; F, El Copal; G, Tirimbina; H, La Selva; I, Veragua; J, Tuba Creek; K, Janirvan; L, San Jorge II. Warmer colors indicate higher elevations.

The large intestine was extracted from 108 birds in Costa Rica and seven in Peru (Table 1, Figure 1, Supplemental Table S1) during fieldwork conducted between May and August 2010 [LSU IACUC protocol 09-001; Sistema Nacional de Areas de Conservación (SINAC) permit number: 109-2010-SINAC; access to genetic material by Comisión Nacional para la Gestión de la Biodiversidad (CONAGEBio) under permit: R010-2010-OT-CONAGEBIO]. Immediately after euthanization, birds were dissected and the largest section of undisturbed large intestine tied off, cut out and deposited into CryoVials. Samples were frozen in liquid nitrogen, following the protocol of Godoy-Vitorino et al. (2010) and kept frozen at the LSUMNS Collection of Genetic Resources until microbial DNA extraction (using Qiagen Power Soil extraction kits). We focused on bird species found in lowland forests across Costa Rica whose ranges extend south into the Peruvian Amazon.

**Table 1:** **Order, family, genus, species, sampling locality, and number of samples used in this study; sampling localities mapped on Figure 1**.

**Order**	**Family**	**Genus**	**Species**	**Sampling locality**											
				***A***	***B***	***C***	***D***	***E***	***F***	***G***	***H***	***I***	***J***	***K***	***L***
Apodiformes	Trochilidae	*Amazilia*	*tzacatl*							1					
Apodiformes	Trochilidae	*Florisuga*	*mellivora*			2				1					1
Apodiformes	Trochilidae	*Phaethornis*	*longirostris*		2	1									
Apodiformes	Trochilidae	*Thalurania*	*colombica*			2									
Apodiformes	Trochilidae	*Threnetes*	*ruckeri*		1					3					
Caprimulgiformes	Caprimulgidae	*Nyctidromus*	*albicollis*									2			
Columbiformes	Columbidae	*Geotrygon*	*montana*			1									
Coraciiformes	Momotidae	*Baryphthengus*	*martii*							1					
Cuculiformes	Cuculidae	*Piaya*	*cayana*									1			
Passeriformes	Cardinalidae	*Cyanocompsa*	*cyanoides*			2				2				3	1
Passeriformes	Cardinalidae	*Habia*	*atrimaxillaris*		1	1									
Passeriformes	Cardinalidae	*Habia*	*fuscicauda*							4					
Passeriformes	Emberizidae	*Arremon*	*aurantiirostris*				1			1					
Passeriformes	Emberizidae	*Arremonops*	*conirostris*			1									
Passeriformes	Formicariidae	*Formicarius*	*analis*		1		1								
Passeriformes	Furnariidae	*Automolus*	*ochrolaemus*	1											
Passeriformes	Furnariidae	*Dendrocincla*	*fuliginosa*							1					
Passeriformes	Furnariidae	*Glyphorynchus*	*spirurus*								1	1			
Passeriformes	Furnariidae	*Xiphorhynchus*	*susurrans*			2				1					
Passeriformes	Icteridae	*Cacicus*	*uropygialis*			2			1						
Passeriformes	Incertae Sedis	*Saltator*	*maximus*							1					
Passeriformes	Parulidae	*Myiothlypis*	*fulvicauda*					1							
Passeriformes	Pipridae	*Manacus*	*aurantiacus*			1									
Passeriformes	Pipridae	*Manacus*	*candei*							6					
Passeriformes	Pipridae	*Ceratopipra*	*mentalis*		1	2	1			3					
Passeriformes	Thamnophilidae	*Cymbilaimus*	*lineatus*										1		
Passeriformes	Thamnophilidae	*Gymnopithys*	*leucaspis*			2				1					
Passeriformes	Thamnophilidae	*Hylophylax*	*naevioides*							1					
Passeriformes	Thamnophilidae	*Microrhopias*	*quixensis*			1			1						
Passeriformes	Thamnophilidae	*Myrmeciza*	*exsul*							1			1		
Passeriformes	Thraupidae	*Chlorophanes*	*spiza*			1									
Passeriformes	Thraupidae	*Oryzoborus*	*funereus*							1					
Passeriformes	Thraupidae	*Ramphocelus*	*costaricensis*			1									
Passeriformes	Thraupidae	*Ramphocelus*	*passerinii*							3					
Passeriformes	Thraupidae	*Sporophila*	*corvina*							1					
Passeriformes	Thraupidae	*Tachyphonus*	*luctuosus*									1			
Passeriformes	Thraupidae	*Tangara*	*gyrola*			1									
Passeriformes	Thraupidae	*Tangara*	*larvata*							1		1			
Passeriformes	Thraupidae	*Thraupis*	*episcopus*							1					
Passeriformes	Thraupidae	*Volatinia*	*jacarina*							1					
Passeriformes	Tityridae	*Tityra*	*inquisitor*			1									
Passeriformes	Troglodytidae	*Cantorchilus*	*nigricapillus*									1			
Passeriformes	Troglodytidae	*Henicorhina*	*leucosticta*							1		1			
Passeriformes	Turdidae	*Turdus*	*grayi*							1					
Passeriformes	Tyrannidae	*Attila*	*spadiceus*	2											
Passeriformes	Tyrannidae	*Elaenia*	*flavogaster*							1					
Passeriformes	Tyrannidae	*Mionectes*	*oleagineus*		1	1				1				1	1
Passeriformes	Tyrannidae	*Myiarchus*	*tuberculifer*			1									
Passeriformes	Tyrannidae	*Myiozetetes*	*granadensis*							1					
Passeriformes	Tyrannidae	*Myiozetetes*	*similis*			3									
Passeriformes	Tyrannidae	*Onychorhynchus*	*coronatus*							1					
Passeriformes	Tyrannidae	*Platyrinchus*	*coronatus*		1										
Passeriformes	Tyrannidae	*Tolmomyias*	*sulphurescens*							1					
Passeriformes	Vireonidae	*Hylophilus*	*flavipes*	1											
Piciformes	Galbulidae	*Galbula*	*ruficauda*							2					
Piciformes	Picidae	*Melanerpes*	*pucherani*							1					
Piciformes	Ramphastidae	*Pteroglossus*	*torquatus*						1						
Trogoniformes	Trogonidae	*Trogon*	*massena*									1			
Trogoniformes	Trogonidae	*Trogon*	*rufus*		1							2			

(A) COSTA RICA: Prov. Puntarenas, Golfito, Parque Nacional Piedras Blancas, elev 75 m. (B) COSTA RICA: Prov. Puntarenas, Osa, Los Charcos, elev 65–110 m. (C) COSTA RICA: Prov. Puntarenas, Osa, Reserva Forestal Golfo Dulce Rd. to Drake, elev 260 m. (D) COSTA RICA: Prov. Puntarenas, Quepos, Londres, elev 200 m. (E) COSTA RICA: Prov. San Jose, Tarrazu, Santa Juana, elev 400 m. (F) COSTA RICA: Prov. Cartago, Tucuttique, Reserva Biologica El Copal, elev 1050 m. (G) COSTA RICA: Prov. Heredia, Sarapiqui, Reserva Biologica, Tirimbina, elev 170 m. (H) COSTA RICA: Prov. Heredia, Sarapiqui, Estacion Biologica La Selva. (I) COSTA RICA: Prov. Limon, Teleferico Veragua, elev 260–430 m. (J) COSTA RICA: Prov. Limon, Talamanca, Tuba Creek, elev 80 m. (K) PERU: Pasco, Prov. Oxapampa, Dist. Puerto Bermudez, Comunidad San Juan, trail to Janirvan, waterfall, elev 325 m. (L) PERU: Junin, Prov. Satipo, Dist. Rio Tambo, Sector Yavyos, Fundo San Jorge II, elev 415 m.

### Bird DNA sequencing and phylogenetic tree estimation

DNA was extracted from the liver, heart or pectoral muscle of birds using the standard tissue protocol from Qiagen DNeasy kits. The mitochondrial locus ND2 was sequenced using the L5215 (5′ TAT CGG GCC CAT ACC CCG AAA AT 3′) and H6313 (5′ CTC TTA TTT AAG GCT TTG AAG GC 3′) primers and a thermocycler program of 94°C for 2 min followed by 35 cycles of 94°C for 60 s, 45°C for 30 s, 72°C for 60 s and a final extension of 7 min at 72°C (Brumfield et al., 2007). Amplicons were cleaned using a BigDye Terminator kit and sequenced on ABI PRISM 3730xl at Beckman Coulter Genomics (Danvers, Massachusetts). The sequences were aligned with MUSCLE (Edgar, 2004) and we used BEAST (Drummond and Rambaut, 2007) to generate a sample from the posterior distribution of phylogenetic trees given the data (partitioned GTR + Γ model with estimated base frequencies). Because many of the internal branches of a multi-order bird phylogeny are short and several genomic-scale datasets have recently resolved some of these common conflicts, we also constrained the tree's internal nodes to reflect current genomic understanding of bird taxonomy (Klicka et al., 2007; Tello et al., 2009; Barker et al., 2013).

### Microbial fingerprint

Following Gloor et al. (2010) we used combinatoric primers and massive multiplexing of PCR amplicons for sequencing on an Illumina Hi-Seq. This method uses paired-end sequencing technology to generate pairs of sequences with 100% overlap across variable region 6 (V6) of the 16S component of rRNA; primer sequences align to positions 967–985 and 1078–1061 on *Escherichia coli* 16S rRNA (Gloor et al., 2010). We chose the V6 region of 16S because it is short enough that the sequencing technology was able to cover the entire region in both directions but variable enough to differentiate bacterial species (Chakravorty et al., 2007).

Several samples were extracted or amplified more than once to quantify differences along the digestive tract and/or determine how sensitive the methods are to differences. One sample was amplified and sequenced a second time from a single extraction; these replicates are *Cyanocompsa.cyanoides.1.1* and *Cyanocompsa.cyanoides*.*1.2.* Three birds had two extractions completed and were sequenced independently: *Attila.spadiceus.1* had one extraction from the posterior large intestine and the other from the anterior large intestine; *Trogon.rufus.2* had two extractions in tandem from the posterior large intestine; *Nyctidromus.albicollis.1* had one extraction from the posterior large intestine and a second from one of the ceca.

We used several measures of sequence quality control. First, both reads of a given pair had to match across 100% of the bases. The pairs also must have no errors in the individual tag or priming sequence. We used the BELLEROPHON (Huber et al., 2004) function within the mothur program (Schloss et al., 2009) to identify and discard potentially chimeric sequences. Finally, we used mothur to discard sequences that did not blast to the domain Bacteria. The reads passing these filters were included in the final dataset.

### Subsampled datasets

To assess patterns across different spatial, taxonomic and ecological scales, we subdivided the dataset eight ways.

1. Full dataset: all samples (*N* = 116).
2. >2 individuals: all individuals belonging to species sampled more than once (*N* = 80, removes singletons).
3. CFM: all individuals from the three species that were sampled from both Costa Rica and Peru: *Cyanocompsa cyanoides, Florisuga mellivora* and *Mionectes oleagineus* (*N* = 17, this dataset allows investigation of large-scale geographic differences between birds of the same species).
4. *Cyanoides*: all individuals belonging to the species *Cyanocompsa cyanoides* (*N* = 8, removes taxonomic variation).
5. Manakins: all individuals from two species that were sampled multiple times and belong to the same family: *Manacus candei* and *Ceratopipra mentalis* (*N* = 13, this dataset allowed us to look at the differentiation between closely related species).
6. Non-passerines: all the birds belonging to orders other than Passeriformes (*N* = 27, removes largest order).
7. Passerines: all the birds belonging to the order Passeriformes (*N* = 88, constrains taxonomic variation to a single order).
8. Tirimbina: all individuals from Tirimbina Biological Reserve, Sarapiquí, Costa Rica, the most densely sampled locality (*N* = 45, removes geographic variation).

### Taxonomic assignment and clustering analyses

The microbial ecology package QIIME (Caporaso et al., 2010b) was used for the following analyses. First, the *de novo* OTU picking protocol was used to assign the reads to phylotypes at 97% sequence similarity because 3% is frequently cited as the “species” level of microbial taxonomy (Schloss and Handelsman, 2005), hereinafter “phylotypes.” Next, we assigned taxonomies to phylotypes using the QIIME implementation of the RDP Classifier 2.2 Program (Wang et al., 2007), with the default confidence threshold of 80%. A “core microbiota” was calculated and included all phylotypes that were found in 100% of the samples.

A pairwise UniFrac (Lozupone and Knight, 2005) distance matrix (UDM) was constructed between each gut microbial community (i.e., each bird specimen). UniFrac distances are calculated based on the amount of branch length in a phylogenetic tree that is unique to either of two environments (vs. how much of the tree is shared by the environments). These distances can be based on presence-absence of OTUs (“unweighted”) or weighted by abundance and our analyses use both, as neither method is agreed to be more appropriate for multi-species microbiota studies. Our data were aligned using the QIIME implementation of PyNAST (Caporaso et al., 2010a) and a microbial phylogenetic tree was constructed with FASTTREE (Price et al., 2009). All individuals were randomly reduced to 3652 reads, equal to the lowest number of reads for any bird in the dataset. Despite this type of rarefaction being inappropriate for some questions (McMurdie and Holmes, 2014), we chose to normalize our data this way because we are comparing “whole microbiome” data across many individuals and highly variable sequencing depths can affect diversity estimates (Goodrich et al., 2014). We constructed UPGMA dendrograms based on both the unweighted UDM and weighted UDM to visually represent the relatedness of the gut microbiota for all datasets. Principal coordinates analysis (PCoA) was also performed on both the weighted and unweighted UniFrac distance matrices.

As a complement to the phylogenetic-based methods, we visualized the data with non-metric multidimensional scaling (NMDS). We square root-transformed the percentage of each sample that belonged to each bacterial phylum, then created a pairwise distance matrix using Bray-Curtis dissimilarity, applied through the VEGDIST function of the VEGAN package (Oksanen et al., 2011) in R (R Development Core Team, 2010). The NMDS function of the ECODIST package (Goslee and Urban, 2007) was then used to calculate the two-dimensional positions of the samples (such that closer samples are more similar), the stress and *R*^2^ value of the plot. Stress values >0.3 should not be considered valid whereas values <0.2 can be considered a good representation of the data (Quinn and Keough, 2002).

### Categorical variable significance

To look for a relationship between categorical variables associated with each bird and the microbial communities, we used the statistical tools Adonis (McArdle and Anderson, 2001) and Anosim (Clarke, 1993) implemented in QIIME. The categorical variables included the American Ornithologists' Union South American Classification Committee's taxonomy, i.e., order, family, genus and species (Remsen et al., 2012), ecological variables, including dietary specialization and habitat (Bennett and Owens, 2002), spatial variables and individual properties, like age (based on percent of skull ossification), stomach contents (e.g., “insects” or “plant material”) and bacterial richness (bacterial taxa identified per bird). Table 2 gives a detailed list of the variables and their sources. We calculated significance of all variables for both the weighted and unweighted UDMs with 999 permutations.

**Table 2:** **Categorical variables tested for significance, including the number of categories within each variable (Cat) and a list of the possible designations (except taxonomic categories)**.

**Variable**	**Cat**	**Description and source**
Order	8	Bird order (Remsen et al., 2012)
Family	24	Bird family (Remsen et al., 2012)
Genus	53	Bird genus (Remsen et al., 2012)
Species	59	Bird species (Remsen et al., 2012)
Diet specific	11	Specific dietary specialization: nectar, generalist, insect, seed, arthropod, fruit, insect/fruit, fruit/insect, nectar/insect, arthropod/vertebrates, fruit/nectar/insects (C. Sanchez, pers. com.)
Diet broad	3	Broad dietary specialization: plant material, animal material, both
Diet B&O	4	Broad dietary specialization assigned by Bennett and Owens (2002): frugivore, nectarivore, insectivore, omnivore
Habitat	5	General habitat assigned by Bennett and Owens (2002): woodland, forest, forest/grassland, forest/grassland/scrub, all habitats
Foraging strata	9	Foraging strata assigned by Stotz et al. (1996): canopy, midstory, understory, terrestrial, under/midstory, midstory/canopy, terrestrial/understory, terrestrial/midcanopy, understory/canopy
Locality	12	Sampling locality (see Figure 1): Tirimbina, Londres, Los Charcos, Piedras Blancas, Golfo Dulce, El Copal, Janirvan, Tuba Creek, San Jorge II, La Selva, Veragua, Santa Juana
Country	2	Country of sampling: Costa Rica, Peru
NSEW	6	Relative location of sampling locality: north-east Costa Rica, midwest Costa Rica, southwest Costa Rica, middle Costa Rica, mideast Costa Rica, Peru
Elevation	13	Elevation of sampling locality: 65, 75, 80, 110, 170, 200, 250, 260, 325, 400, 415, 430, and 1050 m
Sex	3	Sex of the bird: male, female, unknown
Age	14	Percent of skull ossification (a proxy for age of bird): 0, 3, 5, 10, 15, 20, 25, 50, 70, 75, 90, 95, 100, unknown
Stomach contents	12	Contents of stomach at time of collection: insects, seeds, fruit, plant material, seeds/insects, insects/pollen, fruit/insects, insects/plants, seeds/plants, fruit/insects/seeds, empty, unknown
Phyla richness	10	Number of bacterial phyla identified in the gut microbiota fingerprint: 8, 9, 10, 11, 12, 13, 14, 15, 16, 17
Species richness	5	Which fifth the number of 97% OTUs identified in a gut microbiota fingerprint belongs to: 20%ile, 40%ile, 60%ile, 80%ile, 100%ile

After testing the significance of each variable independently, we ran an additional Adonis test on the most frequently significant variables to quantify the amount of variation each variable was responsible for in the context of other variables. We used the full dataset's unweighted and weighted UDMs as input, calculated 999 permutations, and permuted the order of the variables, which can affect the results of the test. Finally, we constrained the analyses to only permute the data within bird orders, as a measure of controlling for taxonomy. We then reran the weighted and unweighted UDMs.

## Results

After initial quality control steps, 9,897,718 pairs of reads remained with no errors in priming sequence, region of overlap or individual tags. Potentially chimeric sequences (1167, 0.01% of reads) were then discarded. A further 3,58,725 sequences that did not align to any sequence within the domain Bacteria (3.6% of reads) were removed; 75% of these discarded reads belonged to 11 individuals. The reads passing these filters were included in the final dataset, totaling 9,537,817 sequences and averaging 82,222 sequences per individual; reads/sample varied by over two orders of magnitude (range: 3652–853,078).

**Figure 2:**
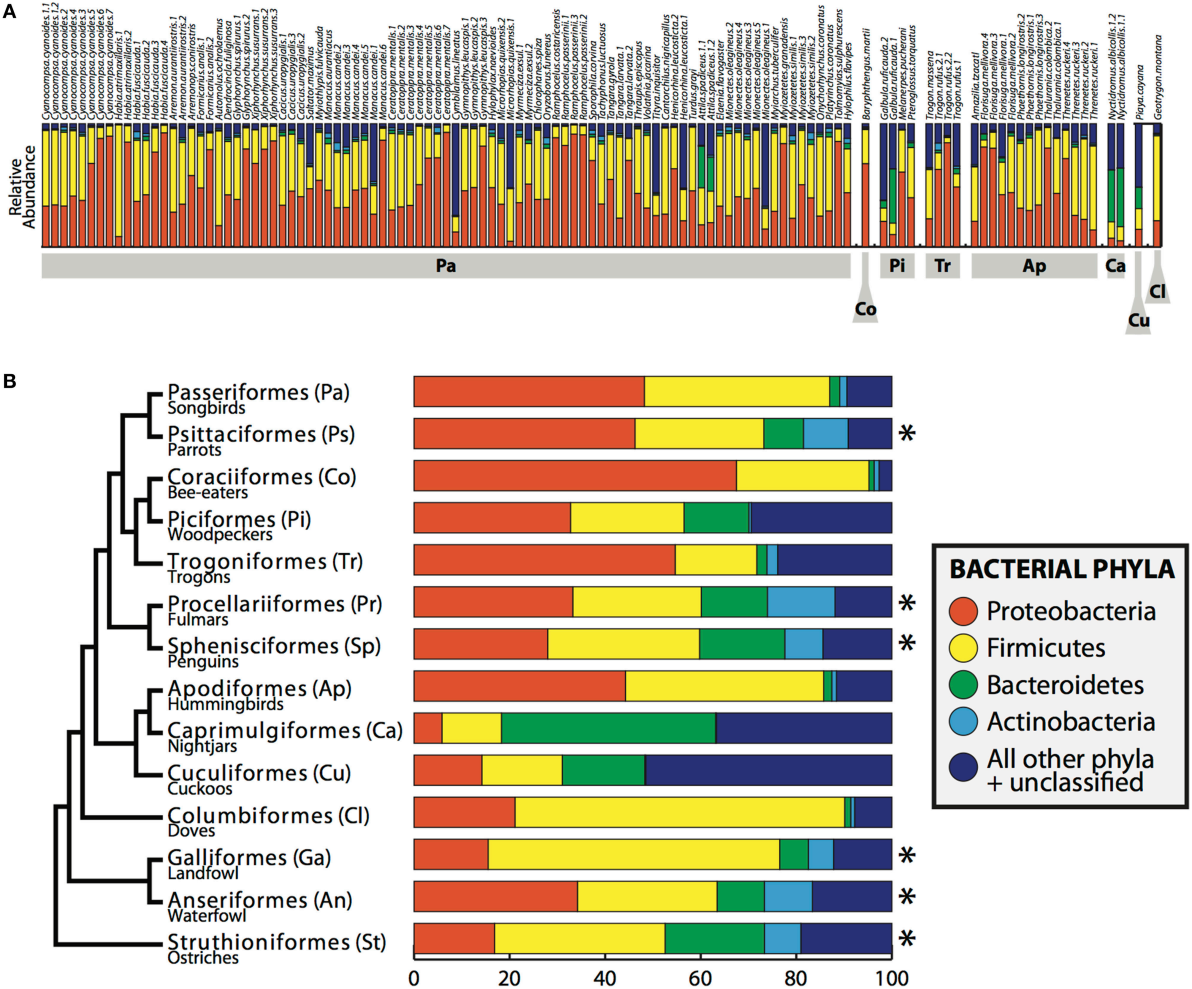
**Bacterial composition of bird gut samples**. **(A)** Relative abundance of bacterial phyla in all birds. Bird orders shown in gray boxes and individuals are labeled across the top. **(B)** Bacterial phyla summarized by bird order, including the individuals in this study and ^*^indicates data obtained from Waite and Taylor (2014). Phylogeny on left is a modified version of the phylogenetic relationship of bird orders from Hackett et al. (2008).

**Figure 3:**
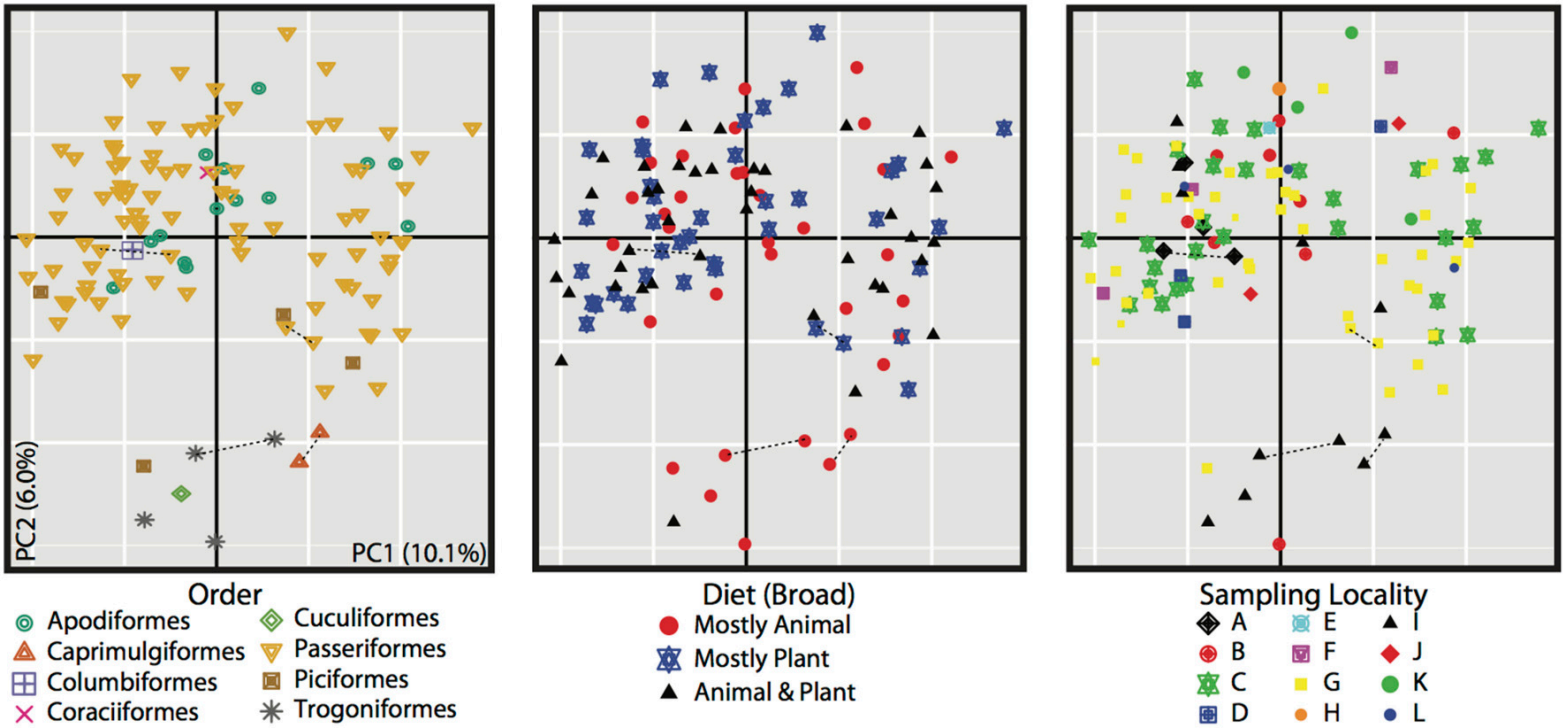
**PCoA of unweighted Unifrac distances for all 116 bird samples**. All three plots are the same, but individuals are colored differently based on metadata, bird order (left), diet (center), and sampling locality (right). Dotted lines connect replicate samples (as described in Materials and Methods).

**Figure 4:**
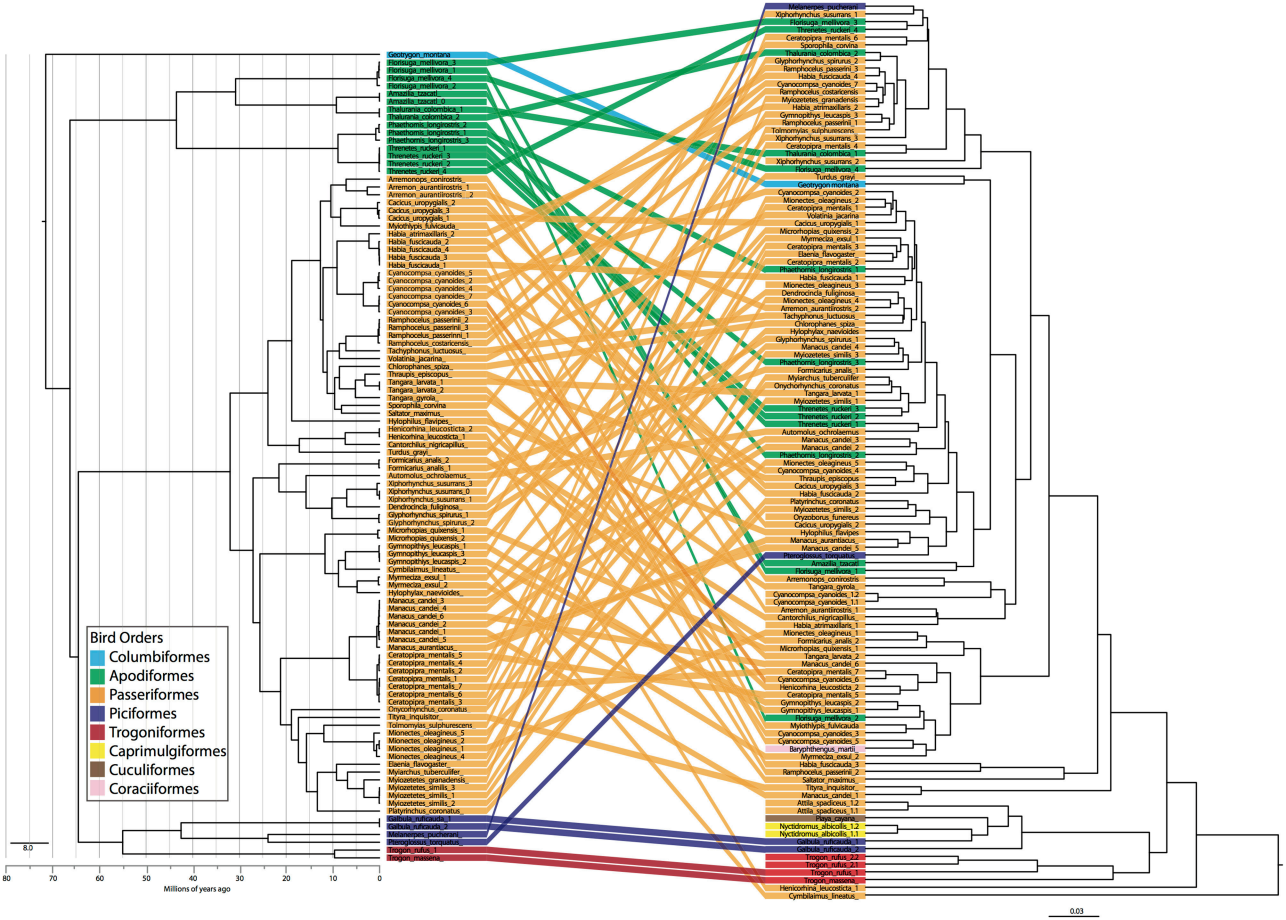
Bird phylogeny (left) obtained using ND2 locus and a constrained species level phylogeny estimated with ^*^BEAST compared to dendrogram of weighted UniFrac distance matrix. Colors indicate bird order and individuals are tracked across the two figures.

Four bacterial phyla were detected in all individuals: Proteobacteria, Firmicutes, Bacteroidetes and Actinobacteria comprising an average of 46.3, 37.3, 3.3, and 1.4% of each sample, respectively (Figure 2). An additional 16 phyla were identified: Acidobacteria, Chlamydiae, Chloroflexi, Cyanobacteria, Deinococcus-Thermus, Fusobacteria, Lentisphaerae, Nitrospira, OD1, OP10, OP11, Planctomycetes, Spirochaetes, TM7, Tenericutes, Verrucomicrobia, with an average of 10.6% of sequences (from each individual) from unknown bacterial phyla. The core microbiota contained 56 phylotypes, 32 of which belonged to 26 known genera (Table 3). An additional 48 phylotypes were detected in >95% of the samples. The number of species-level phylotypes per bird varied between 109 and 288, with an average of 201 (*SD* = 35). Replicate samples were similar to one another in taxonomic composition (Figure 2) and generally clustered close to one another in multivariate space (Figures 3, 4; Figure S2). A heatmap of bacterial phylotypes vs. host taxonomy revealed little clustering and showed how specific phylotypes were found in high abundance in most individuals (Figure S3); most of these phylotypes belonged to the Firmicutes and Proteobacteria.

**Table 3:** **Bacterial taxa identified in 100% of the bird samples (assigned by RDP Classifier Program)**.

**Phylum**		**Class**		**Order**		**Family**		**Genus**	
Actinobacteria	5	Actinobacteria	5	Actinomycetales	4	Corynebacteriaceae	1	*Corynebacterium*	1
						Microbacteriaceae	1		
						Propionibacteriaceae	1	*Propionibacterium*	1
									
									
Bacteroidetes	5	Bacteroidia	1	Bacteroidales	1	Bacteroidaceae	1	*Bacteroides*	1
		Flavobacteria	3	Flavobacteriales	3	Flavobacteriaceae	3	*Chryseobacterium*	1
								*Planobacterium*	1
									
									
Firmicutes	17	Bacilli	12	Bacillales	4	Bacillaceae	2	*Anoxybacillus*	1
									
									
						Staphylococcaceae	1	*Staphylococcus*	1
				Lactobacillales	7	Lactobacillaceae	1	*Lactobacillus*	1
						Leuconostocaceae	2	*Leuconostoc*	1
								*Weissella*	1
									
						Streptococcaceae	3	*Lactococcus*	1
								*Streptococcus*	1
									
									
		Clostridia	4	Clostridiales	4	Clostridiaceae	1	*Clostridium*	1
									
						Veillonellaceae	2	*Veillonella*	1
									
									
Proteobacteria	27	Alpha- proteobacteria	4	Rhizobiales	2	Methylobacteriaceae	1	*Methylobacterium*	1
									
				Sphingomonadales	1	Sphingomonadaceae	1	*Sphingomonas*	1
									
		Beta- proteobacteria	9	Burkholderiales	8	Burkholderiales	2	*Aquabacterium*	1
						incertae sedis		*Tepidimonas*	1
						Comamonadaceae	4	*Acidovorax*	1
								*Diaphorobacter*	1
								*Schlegelella*	1
									
									
						Oxalobacteraceae	1	*Janthinobacterium*	1
									
		Epsilon- proteobacteria	2	Campylobacterales	2	Campylobacteraceae	1	*Campylobacter*	1
						Helicobacteraceae	1	*Helicobacter*	1
		Gamma- proteobacteria	11	Aeromonadales	1	Aeromonadaceae	1	*Aeromonas*	1
				Enterobacteriales	4	Enterobacteriaceae	4	*Escherichia*	1
								*Kluyvera*	1
								*Yersinia*	1
									
				Pseudomonadales	3	Moraxellaceae	2	*Acinetobacter*	1
								*Enhydrobacter*	1
						Pseudomonadaceae	1	*Pseudomonas*	1
				Xanthomonadales	2	Xanthomonadaceae	2	*Stenotrophomonas*	1
									
									
									
									

Gray boxes indicate the phylotype did not align to any named taxa at that taxonomic rank. Numbers in boxes are the total number of identified phylotypes in that group. Full length gray bar at bottom indicates a phylotype that did not align to any named phylum.

### Clustering analyses

The PCoA for the unweighted UDM displayed clustering by taxonomic order (Figure 3), but little obvious clustering by foraging stratum, diet or sampling locality. Host order displayed the most clustering in the weighted UDM PCoA and NMDS plot as well (Figure S1), although little correspondence is seen between the actual bird phylogeny and a dendrogram of the weighted UDM (Figure 4; see Figure S2 for unweighted UDM and Bray-Curtis dendrograms).

### Categorical variable significance

**Figure 5:**
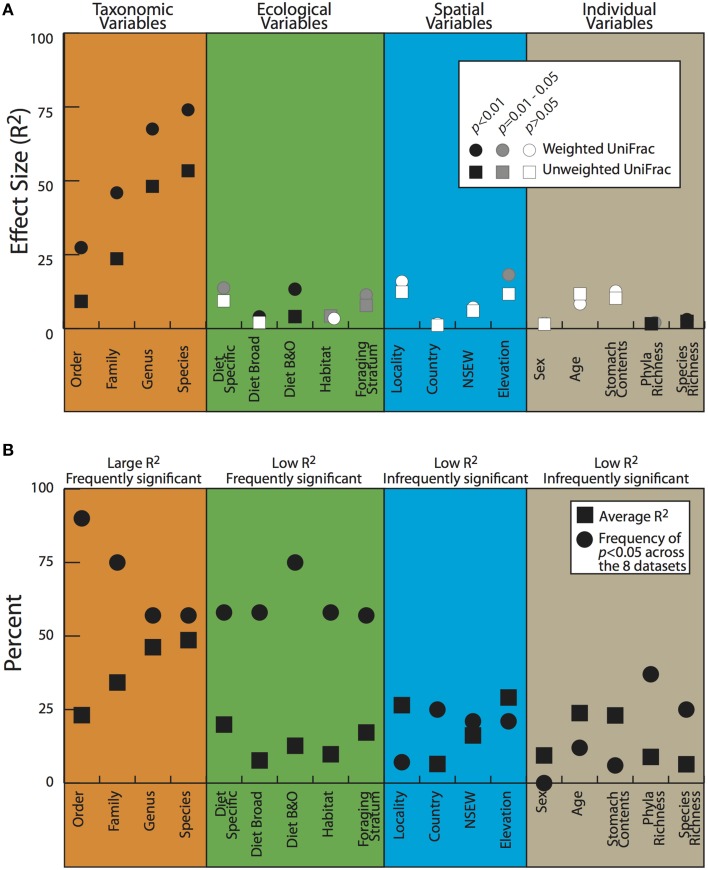
**Adonis test results for the full dataset (A) and averages for all the datasets (B)**. Details on variables in Table 2; datasets described in Materials and Methods.

To look for correlations between categorical variables and the gut microbiota, we conducted two statistical tests for significance on both the weighted and unweighted UDMs (Table 4). For the full dataset, taxonomic categories explained the most amount of variation and were all significant at *p* < 0.05 for the Adonis statistical tests (Figure 5A, Table 4). All other variables explained much less variation although some were significant. The taxonomic categories were also significant in the Anosim test of the full dataset, whereas none of the ecological or spatial variables were (Table 4). Averaging across all 8 datasets, taxonomic variables were the most frequently significant; all four categories (order, family, genus, species) were significant in over 50% of the tests. The taxonomic categories also accounted for the most variation (Figure 5B). The ecological variables were significant in most datasets but generally explained much less variation than the taxonomic variables. The locality and individual variables had generally low *R*^2^ values and were all significant in less than 33% of the tests.

**Table 4:** **Full results for Adonis and Anosim tests of categorical significance**.

**A.**		**Order**				**Family**				**Genus**				**Species**			
		**Adonis**		**ANOSIM**		**Adonis**		**ANOSIM**		**Adonis**		**ANOSIM**		**Adonis**		**ANOSIM**	
		***R*^2^**	***P***	***R***	***P***	***R*^2^**	***P***	***R***	***P***	***R*^2^**	***P***	***R***	***P***	***R*^2^**	***P***	***R***	***P***
Full dataset	Unweighted	9.2	**0.001**	0.136	**0.027**	23.63	**0.001**	0.117	**0.001**	48.12	**0.001**	0.122	**0.022**	53.4	**0.001**	0.133	**0.022**
	Weighted	27.39	**0.001**	0.257	**0.001**	45.95	**0.001**	0.216	**0.001**	67.53	**0.001**	0.250	**0.001**	74.02	**0.001**	0.294	**0.001**
>2 individuals/species	Unweighted	8.11	**0.001**	0.117	0.058	20.53	**0.001**	0.121	**0.009**	33.31	**0.001**	0.121	**0.019**	34.65	**0.001**	0.143	**0.01**
	Weighted	35.33	**0.001**	0.268	**0.002**	47.33	**0.001**	0.239	**0.001**	61.74	**0.001**	0.258	**0.001**	62.92	**0.001**	0.273	**0.001**
CFM	Unweighted	8.08	**0.03**	0.342	**0.016**	14.71	**0.027**	0.317	**0.004**	14.71	**0.027**	0.317	**0.003**	14.71	**0.02**	0.317	**0.003**
	Weighted	8.78	0.199	−0.066	0.599	15.05	0.272	−0.026	0.524	15.05	0.28	−0.026	0.559	15.05	0.277	−0.026	0.568
Cyanoides	Unweighted																
	Weighted																
Manakins	Unweighted									12.08	0.059	0.103	0.125	12.08	0.065	0.103	0.121
	Weighted									13.58	0.156	0.070	0.165	13.58	0.158	0.070	0.183
Non-passerines	Unweighted	31.9	**0.001**	0.504	**0.001**	39.5	**0.001**	0.520	**0.001**	56.22	**0.001**	0.718	**0.001**	59.86	**0.001**	0.707	**0.001**
	Weighted	60.94	**0.001**	0.726	**0.001**	75.5	**0.001**	0.782	**0.001**	81.59	**0.001**	0.561	**0.001**	86.33	**0.001**	0.648	**0.001**
Passerines	Unweighted					16.81	0.196	0.016	0.352	44.56	0.222	0.011	0.459	50.66	0.122	0.033	0.307
	Weighted					24.29	**0.02**	0.059	0.109	53.85	**0.03**	0.098	0.094	59.29	**0.037**	0.108	0.103
Tirimbina	Unweighted	8.58	**0.022**	0.239	**0.014**	28.83	0.685	−0.042	0.711	64.05	0.394	0.029	0.396	64.05	0.386	0.029	0.423
	Weighted	33.08	**0.017**	0.221	0.066	59.17	**0.011**	0.127	0.067	80.69	0.096	0.143	0.163	80.69	0.104	0.143	0.139

**Table:** 

**B.**		**Diet specific**				**Diet broad**				**Diet B&O**				**Habitat**				**Foraging stratum**			
		**Adonis**		**ANOSIM**		**Adonis**		**ANOSIM**		**Adonis**		**ANOSIM**		**Adonis**		**ANOSIM**		**Adonis**		**ANOSIM**	
		***R*^2^**	***P***	***R***	***P***	***R*^2^**	***P***	***R***	***P***	***R*^2^**	***P***	***R***	***P***	***R*^2^**	***P***	***R***	***P***	***R*^2^**	***P***	***R***	***P***
Full dataset	Unweighted	9.36	0.127	0.010	0.401	2.05	0.13	0.020	0.082	4.06	**0.001**	0.000	0.463	4.28	**0.017**	0.034	0.089	7.85	**0.026**	0.050	0.1
	Weighted	13.74	**0.023**	0.002	0.46	3.95	**0.006**	0.064	**0.001**	13.33	**0.001**	0.048	0.153	4.42	0.186	−0.012	0.613	11.39	**0.019**	0.103	**0.024**
>2 individuals/species	Unweighted	13.17	**0.001**	0.106	**0.007**	3.72	**0.007**	0.076	**0.004**	6.24	**0.001**	0.092	**0.016**	5.99	**0.001**	0.077	**0.009**	7.33	0.621	0.021	0.345
	Weighted	16.28	**0.008**	0.051	0.13	5	**0.031**	0.146	**0.001**	19.77	**0.001**	0.050	0.166	6.51	**0.029**	0.025	0.196	16.95	**0.001**	0.132	**0.035**
CFM	Unweighted	14.71	**0.016**	0.317	**0.004**	8.08	**0.022**	0.342	**0.029**	14.71	**0.021**	0.317	**0.003**	14.71	**0.023**	0.317	**0.002**	14.71	**0.027**	0.317	**0.007**
	Weighted	15.05	0.311	−0.026	0.557	8.78	0.207	−0.066	0.59	15.05	0.286	−0.026	0.536	15.05	0.308	−0.026	0.547	15.05	0.307	−0.026	0.537
Cyanoides	Unweighted																				
	Weighted																				
Manakins	Unweighted																	12.08	**0.049**	0.103	0.121
	Weighted																	13.58	0.153	0.070	0.201
Non-passerines	Unweighted	33.77	**0.001**	0.428	**0.001**	13.43	**0.001**	0.359	**0.001**	19.11	**0.001**	0.445	**0.001**	14.53	**0.001**	0.475	**0.001**	27.87	**0.001**	0.285	**0.004**
	Weighted	53.27	**0.003**	0.451	**0.001**	29.07	**0.001**	0.358	**0.001**	45.14	**0.002**	0.642	**0.001**	28.4	**0.001**	0.5321	**0.001**	42.98	**0.005**	0.358	**0.001**
Passerines	Unweighted	8.95	0.073	0.012	0.336	2.55	0.201	0.002	0.407	1.59	**0.046**	0.043	0.085	2.88	0.067	0.033	0.105	8.76	0.109	0.010	0.404
	Weighted	13.25	**0.021**	0.018	0.308	4.61	**0.033**	0.017	0.177	4.88	**0.001**	0.048	0.074	5.62	**0.006**	0.001	0.472	10.84	0.111	−0.036	0.736
Tirimbina	Unweighted	21.88	0.131	0.048	0.214	5.31	0.113	0.048	0.141	4.61	0.387	−0.034	0.718	7.14	0.293	0.062	0.119	17.68	0.082	0.057	0.225
	Weighted	26.38	0.188	0.108	0.082	6.75	0.171	0.127	**0.005**	4.23	0.451	0.040	0.244	9.11	0.204	0.056	0.178	35	**0.05**	0.052	0.267

**Table:** 

**C.**		**Locality**				**Country**				**NSEW**				**Elevation**			
		**Adonis**		**ANOSIM**		**Adonis**		**ANOSIM**		**Adonis**		**ANOSIM**		**Adonis**		**ANOSIM**	
		***R*^2^**	***P***	***R***	***p***	***R*^2^**	***P***	***R***	***P***	***R*^2^**	***P***	***R***	***P***	***R*^2^**	***P***	***R***	***P***
Full dataset	Unweighted	12.36	0.068	0.019	0.324	1.12	0.067	−0.013	0.533	5.95	0.052	0.033	0.183	11.7	0.249	0.008	0.381
	Weighted	15.94	0.072	0.091	**0.049**	1.64	0.09	−0.109	0.749	7.13	0.135	0.081	**0.027**	18.2	**0.013**	0.071	0.151
>2 individuals/species	Unweighted	15.19	0.078	−0.023	0.611	2.17	**0.011**	0.156	0.09	7.89	**0.014**	0.070	0.07	15.48	0.356	−0.056	0.794
	Weighted	18.8	0.092	0.055	0.211	1.57	0.253	−0.069	0.653	8.52	0.137	0.064	0.119	23.36	**0.02**	−0.006	0.521
CFM	Unweighted	33.14	0.105	0.132	0.133	7.22	0.099	0.104	0.151	14.29	**0.044**	0.135	0.071	38.77	0.189	0.232	**0.047**
	Weighted	30.15	0.52	−0.015	0.532	14	**0.048**	0.056	0.229	23.55	0.054	0.107	0.125	34.47	0.6	−0.089	0.725
Cyanoides	Unweighted	58.83	0.238	0.096	0.368	15.47	0.206	0.146	0.169	29.59	0.253	−0.050	0.504	58.83	0.253	0.125	0.347
	Weighted	74.9	0.156	0.165	0.26	26.9	0.172	0.031	0.382	43.34	0.213	0.063	0.293	74.9	0.17	0.250	0.243
Manakins	Unweighted	16.38	0.397	0.202	0.206					16.38	0.399	0.169	0.207	23.93	0.532	0.202	0.215
	Weighted	14.56	0.523	−0.096	0.652					14.56	0.508	−0.080	0.619	15.5	0.822	−0.096	0.636
Non-passerines	Unweighted	22.93	**0.008**	0.163	**0.026**	3.38	0.909	−0.278	0.889	19.12	**0.009**	0.189	**0.018**	24.91	0.101	0.055	0.238
	Weighted	26.36	0.125	0.094	0.121	1.47	0.671	−0.271	0.81	23.33	0.098	0.197	**0.016**	37.53	**0.027**	0.132	0.106
Passerines	Unweighted	13.78	0.504	−0.036	0.755	1.8	**0.019**	0.218	**0.039**	6.53	0.749	−0.028	0.72	13.76	0.5	−0.049	0.77
	Weighted	18.05	0.111	−0.011	0.536	2.13	0.133	−0.039	0.58	7.58	0.312	−0.029	0.676	17.13	0.151	−0.040	0.714
Tirimbina	Unweighted																
	Weighted																

**Table:** 

**D.**		**Sex**				**Age**				**Stomach contents**				**Phyla richness**				**Species richness**			
		**Adonis**		**ANOSIM**		**Adonis**		**ANOSIM**		**Adonis**		**ANOSIM**		**Adonis**		**ANOSIM**		**Adonis**		**ANOSIM**	
		***R*^2^**	***P***	***R***	***P***	***R*^2^**	***P***	***R***	***P***	***R*^2^**	***P***	***R***	***P***	***R*^2^**	***P***	***R***	***P***	***R*^2^**	***P***	***R***	***P***
Full dataset	Unweighted	1.49	0.894	−0.009	0.651	11.75	0.223	−0.027	0.644	10.27	0.095	0.036	0.198	1.63	**0.003**	0.055	**0.046**	2.43	**0.001**	0.0541	**0.003**
	Weighted	1.76	0.441	0.020	0.206	8.37	0.878	−0.133	0.966	12.64	0.118	0.114	**0.016**	2.52	**0.011**	0.030	0.187	3.03	**0.001**	0.025	**0.016**
>2 individuals/species	Unweighted	2.36	0.644	−0.010	0.603	12.92	**0.034**	0.050	0.209	12.25	0.112	0.083	0.064	1.51	0.173	0.086	**0.024**	1.69	0.064	−0.007	0.621
	Weighted	3.04	0.292	0.036	0.171	12.31	0.354	−0.070	0.772	15.82	0.104	0.095	0.084	2.24	0.121	0.002	0.463	2.33	0.095	0.011	0.237
CFM	Unweighted	10.26	0.997	−0.173	0.975	46.68	**0.029**	0.300	**0.047**	31.49	0.415	0.154	0.168	6.56	0.303	−0.177	0.913	6.13	0.507	−0.010	0.487
	Weighted	8.89	0.679	−0.071	0.715	58.21	0.071	0.156	0.194	31	0.45	−0.054	0.592	12.27	0.106	0.024	0.446	7.39	0.335	−0.065	0.707
Cyanoides	Unweighted	32.11	0.086	0.275	0.152	30.75	0.163	0.059	0.312	42.3	0.546	0.200	0.25	13.37	0.652	0.577	0.117	15.81	0.18	0.167	0.321
	Weighted	36.45	0.271	−0.076	0.558	50.63	0.076	0.241	0.132	56.88	0.223	0.183	0.266	34.31	0.088	0.346	0.239	16.72	0.317	−0.076	0.609
Manakins	Unweighted	17.81	0.283	0.087	0.249	16.48	0.365	−0.240	0.879	15.82	0.53	−0.188	0.805	13.88	**0.029**	0.323	**0.016**	10.2	0.129	0.157	0.124
	Weighted	8.73	0.874	−0.196	0.972	16.69	0.413	−0.135	0.676	23.17	0.202	0.301	0.072	27.23	**0.015**	0.179	0.141	10.77	0.253	0.123	0.186
Non-passerines	Unweighted	8.43	0.18	0.073	0.153	33.04	0.058	0.114	0.155	32.32	0.149	0.033	0.318	4.46	0.178	0.048	0.297	4.65	0.119	0.038	0.291
	Weighted	7.14	0.459	0.121	0.069	30.9	0.47	−0.163	0.925	38.05	0.166	0.173	0.055	8.12	0.099	0.068	0.224	8.61	0.083	−0.012	0.493
Passerines	Unweighted	1.81	0.959	−0.043	0.926	11.4	0.523	−0.032	0.655	12.21	**0.014**	0.029	0.283	2.14	**0.002**	0.034	0.15	3.17	**0.001**	0.093	**0.001**
	Weighted	2.05	0.531	−0.028	0.763	12.91	0.303	0.025	0.384	10.05	0.501	0.055	0.199	5.86	**0.001**	0.043	0.127	4.65	**0.001**	0.021	0.169
Tirimbina	Unweighted	5.05	0.177	−0.076	0.905	17.02	0.81	−0.187	0.983	17.29	0.795	0.037	0.358	3.26	**0.025**	0.057	0.174	3.11	0.056	0.024	0.262
	Weighted	4.43	0.444	−0.037	0.693	11.66	0.783	−0.057	0.7	7.8	0.93	0.118	0.132	3.69	0.156	−0.028	0.639	1.91	0.478	0.051	0.097

Effect size (R^2^ or R) and significance shown; results for inappropriate tests are grayed out. P-values less than 0.05 highlighted in pink and bolded.

Using the “Tirimbina” dataset, which included all individuals from a single locality, host order was still significant, with other taxonomic levels less so (Table 4). Conversely, in our most taxonomically restricted dataset including a single species, “*Cyanoides*,” geographic variables explained a large amount of variation but the effect was not significant (unweighted UDM *R*^2^ = 58%, *p* = 0.23; weighted UDM *R*^2^ = 74.9%, *p* = 0.156).

The multifactor Adonis tests yielded different results for the weighted and unweighted UniFrac distance matrices. Foraging stratum, host order and bacterial richness (at the phylum level) were all significant (*p* < 0.05) for the unweighted UDM and accounted for around 10% of the variation each (Table 5A). No variables were significant for the weighted UDM (Table 5B). When data were permuted within the taxonomic orders (i.e., controlling for high-level host taxonomy), the significance of the variables did not change (Tables 5C,D), although the amount of variation that the host taxonomy explained increased.

**Table 5:** **Multifactorial Adonis tests for categorical significance**.

	***R*^2^**	***p***
**A. UNWEIGHTED UDM**		
Diet	0.01161	0.653
Foraging stratum	0.11111	**0.007**
Locality	0.08343	0.426
Host order	0.10287	**0.003**
Bacterial richness	0.10607	**0.029**
**B. WEIGHTED UDM**		
Diet	0.00688	0.764
Foraging stratum	0.09983	0.095
Locality	0.13718	0.064
Host order	0.07614	0.184
Bacterial richness	0.06742	0.497
**C. UNWEIGHTED UDM—CONTROLLING FOR HOST ORDER**		
Diet	0.01161	0.806
Foraging stratum	0.11111	**0.008**
Locality	0.08343	0.695
Host family	0.24009	**0.014**
Bacterial richness	0.10261	**0.025**
**D. WEIGHTED UDM—CONTROLLING FOR HOST ORDER**		
Diet	0.00688	0.766
Foraging stratum	0.09983	0.246
Locality	0.13718	0.093
Host family	0.21724	0.304
Bacterial richness	0.04818	0.833

P-values less than 0.05 are bolded.

## Discussion

Microorganisms are an essential component of biodiversity, and vertebrate evolutionary history is incomplete without an adequate understanding of our microbiota. This study greatly increases our basic understanding of what organisms live in the guts of wild Neotropical birds, spanning eight orders within Aves. We found taxonomic profiles similar to other avian gut studies with some exceptions. Compared to the meta-analysis performed by Waite and Taylor (2014), our samples appear to be enriched for Proteobacteria and have a deficit of Actinobacteria. If this result is not a biological signal, the discrepancy may be due to the difference in marker choice or methods between our study and those in the meta-analysis or to an underlying methodological bias.

Several gut-associated taxa were defined as our “core microbiome.” The phyla Firmicutes, Proteobacteria, Actinobacteria, and Bacteroidetes were found in all our samples and are often found in gut habitats across vertebrates (e.g., Turnbaugh et al., 2008; Roeselers et al., 2011); the same is true for the genera *Bacteroides, Clostridium*, and *Lactobacillus*. Additionally, some genera frequently found in avian guts specifically were identified in all our samples: *Streptococcus* and *Campylobacter* (Zhu et al., 2002; Xenoulis et al., 2010; Videnska et al., 2014). Although several known contaminants of microbiota studies (Salter et al., 2014) were found in our dataset (e.g., *Corynebacterium, Propionibacterium, Streptococcus*), the high biomass of our initial samples (intestinal contents) and the corroboration of many of these genera being found in other gut studies leads us to believe our results are not due to laboratory contamination. Overall, these results suggest that there may be a conserved set of microorganisms found in the guts of birds and perhaps all vertebrates.

### The importance of host taxonomy, ecology, and geographic space

The statistical tests applied above indicate varying degrees of importance for taxonomy, ecology, geographic space, and individual traits for structuring the gut microbiota of birds. Generally speaking, it appears that *who* a bird is most important, *how* a bird lives is possibly important and *where* a bird lives may be of little importance.

Instead of phylogenetic distance, we used taxonomic categories as our evolutionary units, so that we could apply the same statistical methods to all metadata associated with the bird samples. Focusing on hierarchical taxonomic categories also allowed us to overcome the analytical problems associated with high individual variation in gut microbiota (e.g., Turnbaugh et al., 2008; Figure 4) while still learning about the effect of evolutionary history on the microbiota. Taxonomic categories were most frequently significant in our analyses and explained the most variation. There is clear evidence for associations between gut microbiota and host taxonomy, which has been widely noted in other systems and at varying scales (e.g., Ochman et al., 2010). It is important to note, though, that taxonomy is not the same as phylogeny; our results show that individuals of the same species/genus/family/order are more similar to one another than they are to individuals in other groups, not that more closely related species/genera/families/orders have more similar microbiota. In fact, little correlation was seen between the individual phylogeny and the gut microbiota dendrogram (Figure 4). Does this mean that phylogeny is not an underlying factor shaping the gut microbiota? We believe that it would be difficult to get significant signal at all taxonomic levels without phylogeny playing some role but this requires further investigation that may require parsing the microbiome into vertically vs. horizontally transmitted bacteria. It would not be surprising to find some vertical transmission of microbiota from parent to offspring occurring in birds, as all the birds in this study feed their young by regurgitating food into the mouth of nestlings. The exact role of evolutionary history on gut microbiota is an exciting next step.

We also found small but significant associations between the gut microbiota and host ecology. Broad dietary classifications (e.g., mostly plant, mostly animal, plant/animal) were more significant than specific dietary specializations and the literal stomach contents were only significant in two of the 32 tests. Perhaps long term habits or nutritional content have greater influence on gut microbiota than day to day food intake, which is consistent with other studies showing the stability of the avian gut community once established (Benskin et al., 2010) and that diet can drive the convergence of gut microbiota in non-avian vertebrates (Ley et al., 2008a,b; Muegge et al., 2011). Of methodological note: the “stomach contents” variable contains a lot of variance particularly with respect to specificity and accuracy, as it is recorded in the field and only general data are taken, (i.e., “plant material” or “insects”).

Strata and habitat are important ecological aspects of avian biology. Foraging strata is associated with genetic divergence in Neotropical birds (Burney and Brumfield, 2009; Smith et al., 2014) because ecology affects dispersal ability. Our results reinforce the importance of foraging strata on avian biology and support an important role for ecology in differentiation of both host and microbiota. Microbes from the same ecological niche on the human body are able to share genes on a global scale (Smillie et al., 2011)—perhaps the microbiota of birds that share ecological niches are able to transfer genetic material as well. We could not decipher which aspect of host ecology is directly responsible for the signal we discovered in our current data—is it that birds in the same foraging stratum are exposed to the same environment microbes, that their behavior exposes them to particular microbes within the environment or that similar genes recruit specific microbes? Investigating how these aspects of host ecology affect gut microbiota is an exciting avenue of future research.

Despite ecological factors possibly being important for avian gut microbiota, (external) geographic space showed little effect on the microbiota. All the locality variables had poor correlations with the gut microbiota and our spatial tests revealed no statistical significance between space and microbiota. Thus, bird gut microbiota are likely not just a random assortment of the microbes available in a given environment. Can we further conclude that physical space has little effect on the microbiota? Possibly. None of our analyses found that gut communities that are spatially closer are more closely related, both across Aves and within a single species (*Cyanocompsa cyanoides)*. Alternatively, the apparent lack of effect of locality may be an issue of sampling. This dataset contains few species (or even genera) with multiple individuals. If locality is important, we might expect it to be working within species instead of across higher taxonomic levels. Hird et al. (2014) found that locality was significantly correlated with gut microbiota within 34 brown-headed cowbirds from two localities. Scale of analysis may be critical for detecting what factors are contributing to divergence and much more work is needed to define appropriate geographic, taxonomic and ecological sample sizes. Regardless, it appears that geographic space is much less important than taxonomy and ecology for structuring the gut microbiota in birds.

### Incorporating microbiome studies into collection based research

Wild organisms have different microbiota than captive conspecifics (Wienemann et al., 2011) and microbial communities change once the host organism has died (Hauther et al., 2015). Thus, field preservation of the microbiota generates unique and irretrievable data. Studying the microbiota informs us about the host but in a comparative framework, and we can learn about evolutionary processes above the individual as well (e.g., Ley et al., 2008b). The data for this paper were obtained without additional sampling effort; every result represents information that would have been lost had we not collected the intestinal contents for this purpose. The methodologies herein can relatively easily be incorporated into field protocols, when vertebrate specimens are being prepared for preservation in research collections.

The addition of microbial fingerprints to studies of natural history and evolution provides a non-traditional glimpse into some very important biodiversity. Our protocol for field sampling added an average of 5–10 min to specimen preparation time and consisted of isolating the intact intestinal tract, tying an undisturbed portion off at both ends, clipping the intestines on the outside of the ties and storing in liquid nitrogen. Other field researchers may want to include microbiome components in “macrodiversity” studies, even if they cannot process the samples immediately because doing so adds and preserves a novel and frequently entirely unknown dimension of data to vouchered museum specimens.

## Data accessibility

Sequence data, metadata file and an OTU table have been uploaded to FigShare available at figshare.com/s/fe202600737e11e58a2906ec4bbcf141.

## Author contributions

Conception/design of study (SH, RB); field work (CS, SH); acquisition, analysis, and interpretation of data (SH, CS, BC, RB); writing and editing manuscript (SH, CS, BC, RB).

### Conflict of interest statement

The authors declare that the research was conducted in the absence of any commercial or financial relationships that could be construed as a potential conflict of interest.

## Supplementary material

Figure S1 **Principal coordinates analyses on unweighted (left column) and weighted (middle column) UniFrac distances and Non-metric Multidimensional Scaling (NMDS) analysis of bacterial composition of samples (right column)**. Samples are colored by bird order (top row), foraging strata (second row), diet (third row), and sampling locality (bottom row).

Figure S2 **Gut community dendrograms of (A) unweighted UniFrac distance and (B) Bray-Curtis distance**. Colors indicate host bird taxonomic order.

Figure S3 **Heatmap of relative abundance of each identified phylotypes (columns) for each individual (rows)**. Taxonomic class of the bacteria and family of the bird are shown with gray bars. Horizontal black lines delimit taxonomic orders of the birds.

Supplemental Table S1 **Detailed information about each sample, including taxonomic information, dietary assignments, habitat, foraging stratum (FS), sampling locality (L), elevation (Elev), percent of skull ossification (Age), bacterial phyla identified (BRP), the percentage of individuals with fewer phylotypes identified (BRS) and LSUMNS bird tissue collection number (B#)**.
