# Supplementary figures and images for: Comparative Gut Microbiota of 59 Neotropical Bird Species
Source: Front Microbiol. 2015 Dec 21;6:1403. doi: 10.3389/fmicb.2015.01403 (PMC4685052; doi:10.3389/fmicb.2015.01403)

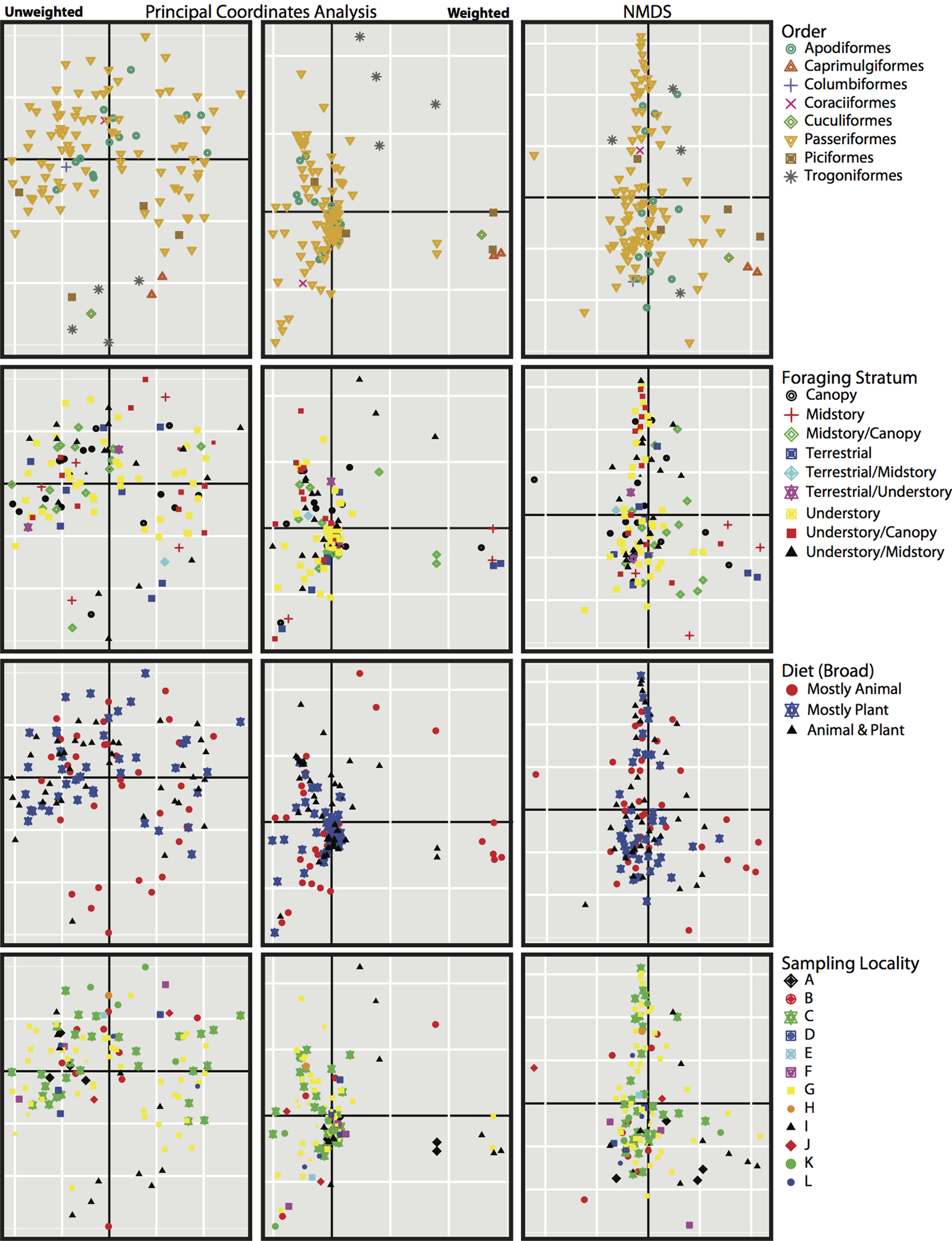

Supplement: Figure S1 — Principal coordinates analyses on unweighted (left column) and weighted (middle column) UniFrac distances and Non-metric Multidimensional Scaling (NMDS) analysis of bacterial composition of samples (right column). Samples are colored by bird order (top row), foraging strata (second row), diet (third row), and sampling locality (bottom row). [file Image1.TIFF]

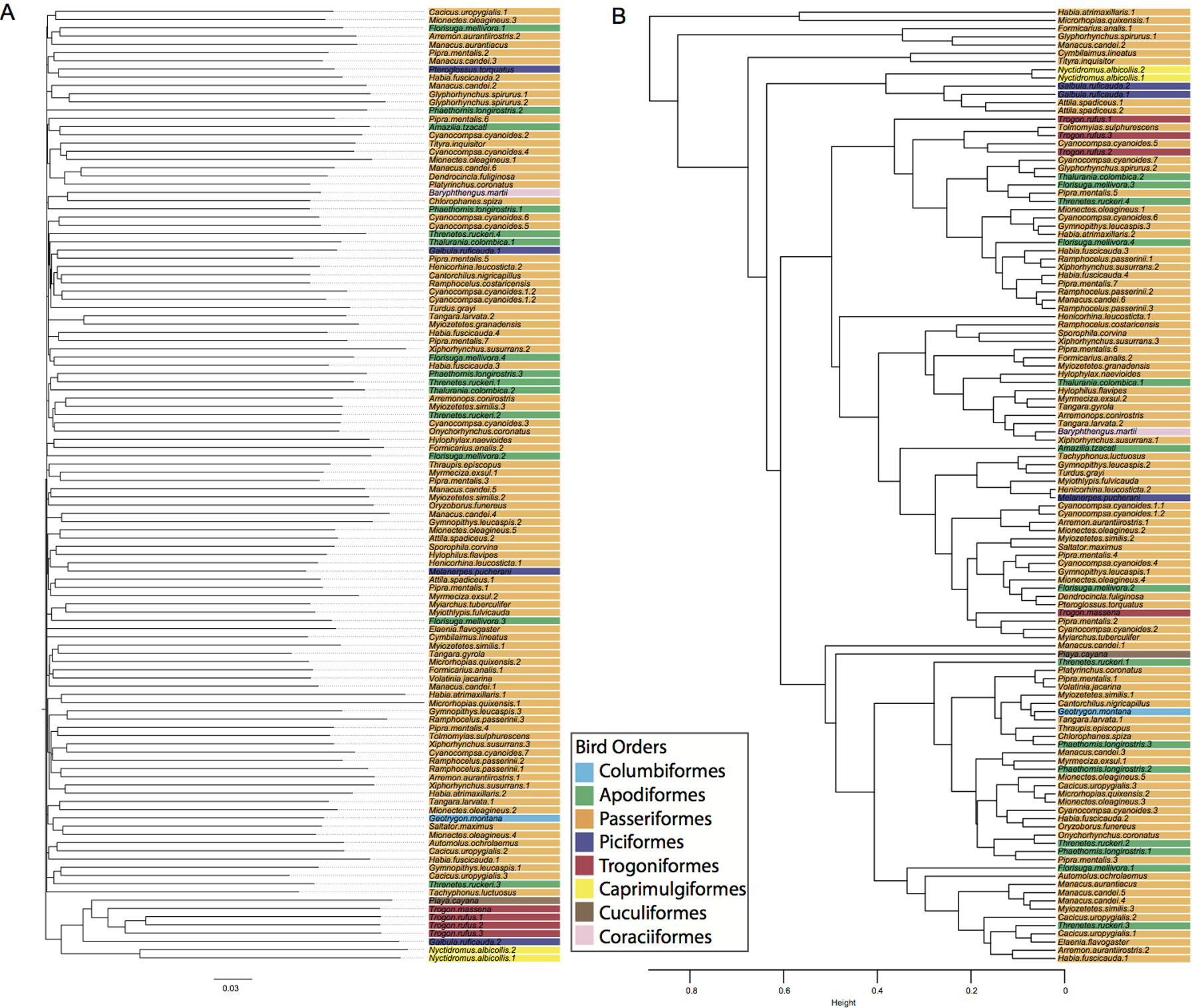

Supplement: Figure S2 — Gut community dendrograms of (A) unweighted UniFrac distance and (B) Bray-Curtis distance. Colors indicate host bird taxonomic order. [file Image2.TIFF]

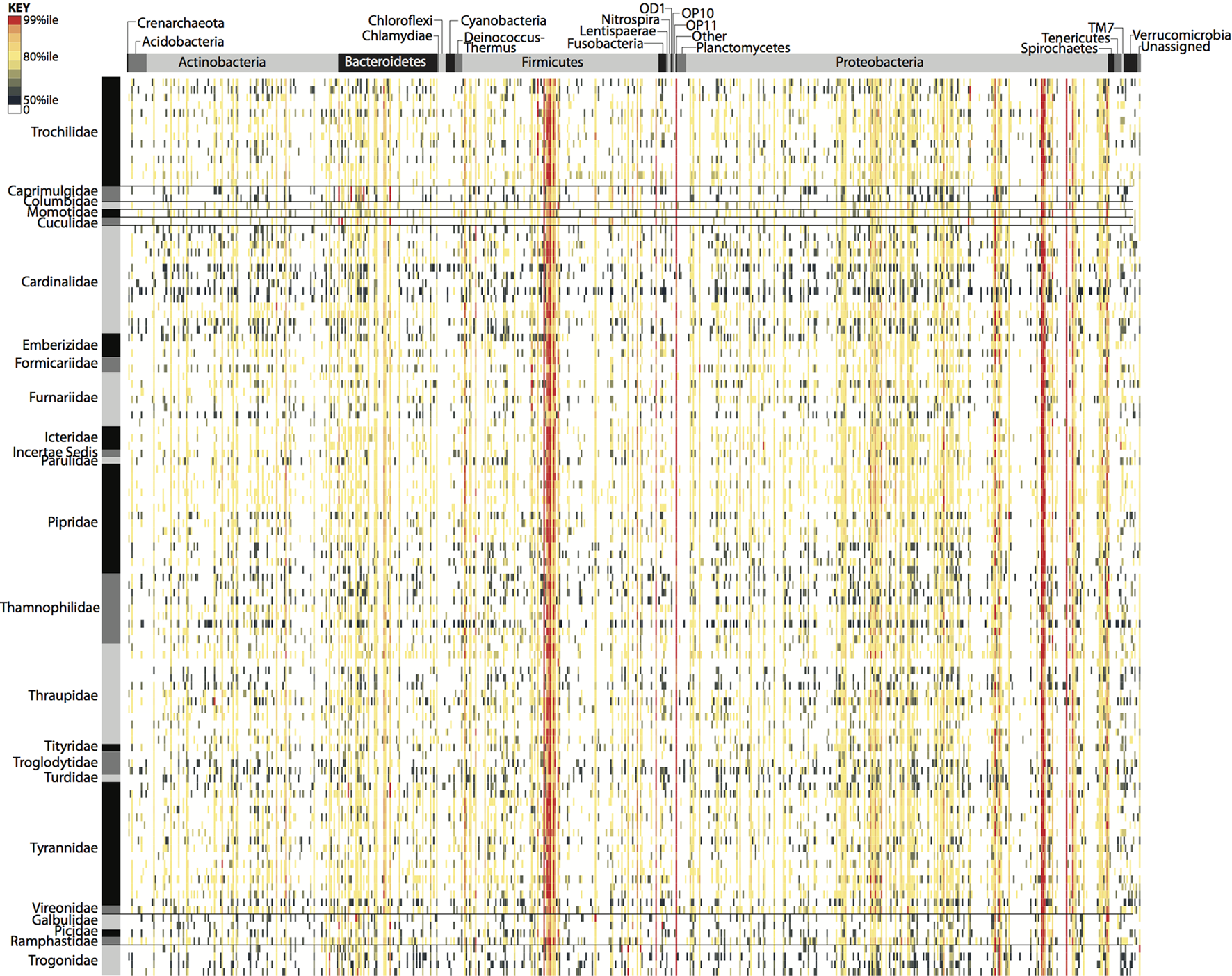

Supplement: Figure S3 — Heatmap of relative abundance of each identified phylotypes (columns) for each individual (rows). Taxonomic class of the bacteria and family of the bird are shown with gray bars. Horizontal black lines delimit taxonomic orders of the birds. [file Image3.TIFF]
