# Supplementary material for: Comparative Gut Microbiota of 59 Neotropical Bird Species
Source: Front Microbiol. 2015 Dec 21;6:1403. doi: 10.3389/fmicb.2015.01403 (PMC4685052; doi:10.3389/fmicb.2015.01403)
Supplement: Supplemental Table S1 — Detailed information about each sample, including taxonomic information, dietary assignments, habitat, foraging stratum (FS), sampling locality (L), elevation (Elev), percent of skull ossification (Age), bacterial phyla identified (BRP), the percentage of individuals with fewer phylotypes identified (BRS) and LSUMNS bird tissue collection number (B#). [file Table1.PDF]

Supplemental Table S1 (Hird et al. 2015): Detailed information about each sample used in Chapter 5, including taxonomic information, dietary assignments, habitat, foraging stratum (FS), sampling locality (L), elevation (Elev), percent of skull ossification (Age), bacterial phyla identified (BRP), the percentage of individuals with fewer phylotypes identified (BRS) and LSUMNS bird tissue collection number (B#).

Genus species (ID)	Order	Family	Diet Specific	Diet Br	Diet BO	Habitat	FS	L	Elev (m)	Age	Stomach Contents	BRP	BRS (%)	Sex	B#
Amazilia tzacatl	Apodiformes	Trochilidae	nectar	h	NE	FT SC GR	U_C	G	170	0	nr	13	100	F	71825
Florisuga mellivora 1	Apodiformes	Trochilidae	nectar insect	o	NE	FT SC GR	M_C	C	260	10	insect	13	20	F	71967
Florisuga mellivora 2	Apodiformes	Trochilidae	nectar insect	o	NE	FT SC GR	M_C	L	415	70	insect pollen	13	80	M	73941
Florisuga mellivora 3	Apodiformes	Trochilidae	nectar insect	o	NE	FT SC GR	M_C	G	170	0	nr	12	60	M	71880
Florisuga mellivora 4	Apodiformes	Trochilidae	nectar insect	o	NE	FT SC GR	M_C	C	260	0	e	12	60	F	71964
Phaethornis longirostris 1	Apodiformes	Trochilidae	nectar	h	NE	FT SC GR	U	C	65	10	e	15	60	F	71984
Phaethornis longirostris 2	Apodiformes	Trochilidae	nectar	h	NE	FT SC GR	U	B	110	3	nr	14	60	M	71935
Phaethornis longirostris 3	Apodiformes	Trochilidae	nectar	h	NE	FT SC GR	U	B	110	0	e	14	60	F	71928
Thalurania colombica 1	Apodiformes	Trochilidae	nectar	h	NE	FT SC GR	U_M	C	65	0	nr	8	20	M	71982
Thalurania colombica 2	Apodiformes	Trochilidae	nectar	h	NE	FT SC GR	U_M	C	65	10	insect	15	60	nr	71985
Threnetes ruckeri 1	Apodiformes	Trochilidae	nectar	h	NE	FT SC GR	U	G	170	20	e	10	20	F	71857
Threnetes ruckeri 2	Apodiformes	Trochilidae	nectar	h	NE	FT SC GR	U	B	110	5	nr	12	80	F	71920
Threnetes ruckeri 3	Apodiformes	Trochilidae	nectar	h	NE	FT SC GR	U	G	170	0	nr	12	40	F	71869
Threnetes ruckeri 4	Apodiformes	Trochilidae	nectar	h	NE	FT SC GR	U	G	170	20	e	9	40	M	71853
Nyctidromus albicollis 1.1	Caprimulgiformes	Caprimulgidae	insect	c	IN	WO	T	I	430	100	insect	12	20	nr	71999
Nyctidromus albicollis 1.2	Caprimulgiformes	Caprimulgidae	insect	c	IN	WO	T	I	430	100	insect	11	40	nr	71999
Geotrygon montana	Columbiformes	Columbidae	seed	h	FR	WO	T	C	65	95	plant	14	40	F	71937
Baryphthengus martii	Coraciiformes	Momotidae	arthropod vert	c	IN	FT	U_M	G	170	100	nr	12	60	F	71827
Piaya cayana	Cuculiformes	Cuculidae	insect	c	IN	WO	C	I	430	100	insect	10	20	M	71998
Cyanocompsa cyanoides 1 1	Passeriformes	Cardinalidae	seed	h	FR	WO	U	G	170	50	e	14	60	F	71872
Cyanocompsa cyanoides 1 2	Passeriformes	Cardinalidae	seed	h	FR	WO	U	G	170	50	e	12	40	F	71872
Cyanocompsa cyanoides 2	Passeriformes	Cardinalidae	seed	h	FR	WO	U	K	325	100	nr	13	100	nr	74234
Cyanocompsa cyanoides 3	Passeriformes	Cardinalidae	seed	h	FR	WO	U	C	260	100	plant	12	40	F	71965
Cyanocompsa cyanoides 4	Passeriformes	Cardinalidae	seed	h	FR	WO	U	K	325	100	seeds	13	100	M	74232
Cyanocompsa cyanoides 5	Passeriformes	Cardinalidae	seed	h	FR	WO	U	L	250	100	plant	17	100	M	73992
Cyanocompsa cyanoides 6	Passeriformes	Cardinalidae	seed	h	FR	WO	U	K	325	5	nr	15	80	M	74155
Cyanocompsa cyanoides 7	Passeriformes	Cardinalidae	seed	h	FR	WO	U	C	65	100	e	11	40	M	71944
Habia atrimaxillaris 1	Passeriformes	Cardinalidae	insect frug	o	FR	WO	U	B	110	100	insect	13	100	F	71918
Habia atrimaxillaris 2	Passeriformes	Cardinalidae	insect frug	o	FR	WO	U	C	65	100	nr	12	40	F	71943
Habia fuscicauda 1	Passeriformes	Cardinalidae	insect frug	o	FR	WO	U	G	170	100	fruit insect	11	40	F	71835
Habia fuscicauda 2	Passeriformes	Cardinalidae	insect frug	o	FR	WO	U	G	170	100	e	10	100	F	71851
Habia fuscicauda 3	Passeriformes	Cardinalidae	insect frug	o	FR	WO	U	G	170	5	nr	14	100	F	71832
Habia fuscicauda 4	Passeriformes	Cardinalidae	insect frug	o	FR	WO	U	G	170	100	insect	12	40	M	71839
Arremon aurantiirostris 1	Passeriformes	Emberizidae	generalist	o	FR	WO	T	D	200	100	seeds insects	15	100	F	71811
Arremon aurantiirostris 2	Passeriformes	Emberizidae	generalist	o	FR	WO	T	G	170	5	insect	11	20	M	71858
Arremonops conirostris	Passeriformes	Emberizidae	generalist	o	FR	WO	T_U	C	65	5	seeds	13	60	F	71995
Formicarius analis 1	Passeriformes	Formicariidae	arthropod	c	IN	FT	T	D	200	100	insect	8	20	M	71809
Formicarius analis 2	Passeriformes	Formicariidae	arthropod	c	IN	FT	T	B	110	50	insect	13	80	M	71933
Automolus ochrolaemus	Passeriformes	Furnariidae	insect	c	IN	FT	U	A	75	100	insect	14	20	nr	72780
Dendrocincla fuliginosa	Passeriformes	Furnariidae	arthropod	c	IN	FT	U_M	G	170	100	e	14	100	M	71846
Glyphorhynchus spirurus 1	Passeriformes	Furnariidae	insect	c	IN	FT	U_M	I	430	100	nr	9	20	M	72001

Glyphorhynchus spirurus 2	Passeriformes	Furnariidae	insect	c	IN	FT	U_M	H		100	nr	14	100	F	71820
Xiphorhynchus susurrans 1	Passeriformes	Furnariidae	arthropod	c	IN	FT	U_C	C	65	90	insect	11	20	M	71993
Xiphorhynchus susurrans 2	Passeriformes	Furnariidae	arthropod	c	IN	FT	U_C	C	65	100	e	13	20	F	71941
Xiphorhynchus susurrans 3	Passeriformes	Furnariidae	arthropod	c	IN	FT	U_C	G	170	100	e	10	20	M	71861
Cacicus uropygialis 1	Passeriformes	Icteridae	generalist	o	FR	WO	C	C	260	100	seeds	11	40	M	71968
Cacicus uropygialis 2	Passeriformes	Icteridae	generalist	o	FR	WO	C	F	1050	100	seeds insects	13	40	nr	72046
Cacicus uropygialis 3	Passeriformes	Icteridae	generalist	o	FR	WO	C	C	260	10	fruit	12	60	M	71969
Saltator maximus	Passeriformes	IncertaeSedis	generalist	o	FR	WO	M_C	G	170	10	nr	12	80	nr	71830
Myiothlypis fulvicauda	Passeriformes	Parulidae	arthropod	c	FR	WO	T	E	400	15	insect	15	80	M	72059
Manacus candei 1	Passeriformes	Pipridae	frug	h	IN	FT GR	U	G	170	100	fruit	14	40	F	71826
Manacus candei 2	Passeriformes	Pipridae	frug	h	IN	FT GR	U	G	170	100	e	11	20	F	71833
Manacus candei 3	Passeriformes	Pipridae	frug	h	IN	FT GR	U	G	170	25	e	14	80	M	71849
Manacus candei 4	Passeriformes	Pipridae	frug	h	IN	FT GR	U	G	170	100	e	13	60	F	71836
Manacus candei 5	Passeriformes	Pipridae	frug	h	IN	FT GR	U	G	170	100	nr	12	60	F	71823
Manacus candei 6	Passeriformes	Pipridae	frug	h	IN	FT GR	U	G	170	100	fruit	12	80	F	71866
Manacus aurantiacus	Passeriformes	Pipridae	frug	h	IN	FT GR	U	C	65	nr	plant	14	80	F	71991
Ceratopipra mentalis 1	Passeriformes	Pipridae	frug	h	IN	FT GR	U_M	D	200	25	nr	13	80	M	71807
Ceratopipra mentalis 2	Passeriformes	Pipridae	frug	h	IN	FT GR	U_M	G	170	100	e	14	80	F	71892
Ceratopipra mentalis 3	Passeriformes	Pipridae	frug	h	IN	FT GR	U_M	C	65	50	e	14	60	nr	71953
Ceratopipra mentalis 4	Passeriformes	Pipridae	frug	h	IN	FT GR	U_M	G	170	100	e	13	40	M	71881
Ceratopipra mentalis 5	Passeriformes	Pipridae	frug	h	IN	FT GR	U_M	G	170	100	e	12	20	F	71875
Ceratopipra mentalis 6	Passeriformes	Pipridae	frug	h	IN	FT GR	U_M	B	110	100	e	11	60	M	71930
Ceratopipra mentalis 7	Passeriformes	Pipridae	frug	h	IN	FT GR	U_M	C	65	100	e	9	20	M	71940
Cymbilaimus lineatus	Passeriformes	Thamnophilidae	insect	c	IN	FT	C	J	80	100	insect	12	100	M	72020
Gymnopithys leucaspis 1	Passeriformes	Thamnophilidae	insect	c	IN	FT	U	C	65	100	e	12	60	F	71957
Gymnopithys leucaspis 2	Passeriformes	Thamnophilidae	insect	c	IN	FT	U	G	170	100	e	14	100	F	71860
Gymnopithys leucaspis 3	Passeriformes	Thamnophilidae	insect	c	IN	FT	U	C	65	100	e	14	40	M	71955
Hylophylax naevioides	Passeriformes	Thamnophilidae	insect	c	IN	FT	U	G	170	100	e	15	80	F	71854
Microrhopias quixensis 1	Passeriformes	Thamnophilidae	insect	c	IN	FT	M	F	1050	10	insect	17	100	nr	72125
Microrhopias quixensis 2	Passeriformes	Thamnophilidae	insect	c	IN	FT	M	C	260	100	insect	13	40	M	71966
Myrmeciza exsul 2	Passeriformes	Thamnophilidae	arthropod	c	IN	FT	U	J	80	50	insect	17	60	F	72021
Myrmeciza exsul 1	Passeriformes	Thamnophilidae	arthropod	c	IN	FT	U	G	170	100	e	15	80	M	71873
Chlorophanes spiza	Passeriformes	Thraupidae	frug nectar insect	o	FR	WO	C	C	260	90	nr	13	60	F	71963
Oryzoborus funereus	Passeriformes	Thraupidae	seed	h	FR	WO	U_M	G	170	10	seeds plant	13	100	M	71856
Ramphocelus costaricensis	Passeriformes	Thraupidae	insect frug	o	FR	WO	U_C	C	65	10	e	12	20	M	72016
Ramphocelus passerinii 1	Passeriformes	Thraupidae	frug insect	o	FR	WO	U_C	G	170	100	e	11	40	F	71844
Ramphocelus passerinii 2	Passeriformes	Thraupidae	frug insect	o	FR	WO	U_C	G	170	5	e	11	60	M	71886
Ramphocelus passerinii 3	Passeriformes	Thraupidae	frug insect	o	FR	WO	U_C	G	170	100	insect	12	20	M	71852
Sporophila corvina	Passeriformes	Thraupidae	seed	h	FR	WO	U_M	G	170	5	seeds	14	80	nr	71862
Tachyphonus luctuosus	Passeriformes	Thraupidae	insect frug	o	FR	WO	M_C	I	430	100	insect	17	100	F	72011
Tangara larvata 1	Passeriformes	Thraupidae	insect frug	o	FR	WO	C	I	430	50	seeds plant	15	80	M	71997
Tangara larvata 2	Passeriformes	Thraupidae	insect frug	o	FR	WO	C	G	170	3	e	12	20	nr	71870
Tangara gyrola	Passeriformes	Thraupidae	insect frug	o	FR	WO	C	C	260	100	seeds plant	11	60	F	71973
Volatinia jacarina	Passeriformes	Thraupidae	seed	h	FR	WO	T_U	G	170	75	e	15	80	F	71841

Thraupis episcopus	Passeriformes	Thraupidae	insect frug	o	FR	WO	C	G	170	3	e	13	40	M	71910
Tityra inquisitor	Passeriformes	Tityridae	insect frug	o	IN	FT GR	C	C	65	100	plant	17	100	F	71954
Cantorchilus nigricapillus	Passeriformes	Troglodytidae	arthropod	c	IN	WO	U	I	260	100	insect	9	20	M	72007
Henicorhina leucosticta 1	Passeriformes	Troglodytidae	arthropod	c	IN	WO	U	I	260	100	insect	15	100	M	72008
Henicorhina leucosticta 2	Passeriformes	Troglodytidae	arthropod	c	IN	WO	U	G	170	100	insect	12	80	F	71837
Turdus grayi	Passeriformes	Turdidae	generalist	o	IN	ALL	T_M	G	170	100	nr	14	60	M	71834
Attila spadiceus 1.1	Passeriformes	Tyrannidae	insect frug	o	IN	FT GR	M_C	A	75	100	insect	15	100	M	72081
Attila spadiceus 1.2	Passeriformes	Tyrannidae	insect frug	o	IN	FT GR	M_C	A	75	100	insect	13	100	M	72081
Elaenia flavogaster	Passeriformes	Tyrannidae	insect frug	o	IN	FT GR	C	G	170	50	e	13	40	F	71877
Mionectes oleagineus 1	Passeriformes	Tyrannidae	frug	h	IN	FT GR	U_C	K	325	20	seeds	13	80	F	74190
Mionectes oleagineus 2	Passeriformes	Tyrannidae	frug	h	IN	FT GR	U_C	L	250	10	e	15	80	F	74000
Mionectes oleagineus 3	Passeriformes	Tyrannidae	frug	h	IN	FT GR	U_C	C	65	25	seeds	12	80	M	71850
Mionectes oleagineus 4	Passeriformes	Tyrannidae	frug	h	IN	FT GR	U_C	B	110	50	e	14	100	M	71932
Mionectes oleagineus 5	Passeriformes	Tyrannidae	frug	h	IN	FT GR	U_C	G	170	25	e	14	40	nr	71867
Myiarchus tuberculifer	Passeriformes	Tyrannidae	insect frug	o	IN	FT GR	M_C	C	65	100	e	15	100	M	72089
Myiozetetes granadensis	Passeriformes	Tyrannidae	insect frug	o	IN	FT GR	C	G	170	100	insect plant	11	20	F	71891
Myiozetetes similis 1	Passeriformes	Tyrannidae	insect frug	o	IN	FT GR	M_C	C	65	75	e	13	80	M	72015
Myiozetetes similis 2	Passeriformes	Tyrannidae	insect frug	o	IN	FT GR	M_C	C	65	75	insect	16	100	F	72014
Myiozetetes similis 3	Passeriformes	Tyrannidae	insect frug	o	IN	FT GR	M_C	C	65	100	e	13	20	M	72013
Onychorhynchus coronatus	Passeriformes	Tyrannidae	insect	c	IN	FT GR	M	G	170	100	e	13	60	nr	71871
Platyrinchus coronatus	Passeriformes	Tyrannidae	arthropod	c	IN	FT GR	U_M	B	110	100	insect	16	100	M	71923
Tolmomyias sulphurescens	Passeriformes	Tyrannidae	insect frug	o	IN	FT GR	C	G	170	50	e	12	80	F	71890
Hylophilus flavipes	Passeriformes	Vireonidae	insect	c	IN	FT	M_C	A	75	100	fruit	16	100	M	72075
Galbula ruficauda 1	Piciformes	Galbulidae	insect	c	IN	FT	M	G	170	100	e	9	20	M	71831
Galbula ruficauda 2	Piciformes	Galbulidae	insect	c	IN	FT	M	G	170	100	nr	13	60	F	71828
Melanerpes pucherani	Piciformes	Picidae	insect frug	o	IN	WO	C	G	170	100	fruit insect seeds	13	40	M	71909
Pteroglossus torquatus	Piciformes	Ramphastidae	frug insect	o	FR	FT	C	F	1050	100	fruit	14	100	nr	72054
Trogon massena	Trogoniformes	Trogonidae	generalist	o	OM	FT	M_C	I	430	100	fruit insect	11	40	F	72010
Trogon rufus 1	Trogoniformes	Trogonidae	insect	c	OM	FT	U_M	B	110	25	seeds	14	60	F	71929
Trogon rufus 2.1	Trogoniformes	Trogonidae	insect	c	OM	FT	U_M	I	260	100	fruit insect	15	80	M	72006
Trogon rufus 2.2	Trogoniformes	Trogonidae	insect	c	OM	FT	U_M	I	260	100	fruit insect	14	40	M	72006

Diet Br: h = mostly plant, o = plant and animal, c = mostly animal; Diet BO: FR = frugivore, IN = invertebrates, OM = omnivore, NE = nectar
Habitat: FT = forest, GR = grassland/steppe/savanah, SC = scrub, WO = woodland; Foraging Strata (FS): T = terrestrial, U = understory, M = midcanopy, C = canopy
Locality: See Figure 1, Table 1; Stomach contents: e = empty, nr = not recorded; Sex: M = male, F = female, nr = not recorded
